# Unsteady stagnation-point flow and heat transfer of a special third grade fluid past a permeable stretching/shrinking sheet

**DOI:** 10.1038/srep24632

**Published:** 2016-04-19

**Authors:** Kohilavani Naganthran, Roslinda Nazar, Ioan Pop

**Affiliations:** 1 School of Mathematical Sciences, Faculty of Science & Technology, Universiti Kebangsaan Malaysia, 43600 UKM Bangi, Selangor, Malaysia; 2 Department of Mathematics, Babeş-Bolyai University, 400084 Cluj-Napoca, Romania

## Abstract

In this paper, the unsteady stagnation-point boundary layer flow and heat transfer of a special third grade fluid past a permeable stretching/shrinking sheet has been studied. Similarity transformation is used to transform the system of boundary layer equations which is in the form of partial differential equations into a system of ordinary differential equations. The system of similarity equations is then reduced to a system of first order differential equations and has been solved numerically by using the bvp4c function in Matlab. The numerical solutions for the skin friction coefficient and heat transfer coefficient as well as the velocity and temperature profiles are presented in the forms of tables and graphs. Dual solutions exist for both cases of stretching and shrinking sheet. Stability analysis is performed to determine which solution is stable and valid physically. Results from the stability analysis depict that the first solution (upper branch) is stable and physically realizable, while the second solution (lower branch) is unstable.

Rheology which relies on the perception that non-Newtonian materials exist in real life, explores the properties of matter determining its behavior towards deformation and flow. Blood, saliva, lubricants, polymer solutions and fresh concrete are typical non-Newtonian fluids that disobey the Newton’s law of viscosity, and since they are highly viscous and do exhibit their significant elastic properties, the studies of non-Newtonian flows have grabbed the opportunity to fill the thirst of vast development in most engineering and industrial processes. Consequently, many non-Newtonian models have been established by researchers such that Fosdick and Rajagopal^1^ and Dunn and Rajagopal^2^. It is well established that second grade fluids exhibit the normal stress effect and do not show the shear-thinning and shear-thickening phenomena which many fluids do^2^. However, third grade fluids are capable of describing such phenomena^1^. Moreover, the equation of motion in a third grade fluid is more complicated than the corresponding equation in a second grade fluid. Non-Newtonian fluids with heat and mass transfer are critically essential in paper making and lubrications with greases industries^3^ and due to its substantial practical implications, many researchers have scrutinized non-Newtonian models in various conditions and can be found in numerous literatures by Rajagopal *et al*.^4^, Pakdemirli^5^, Makinde^6^, Vajravelu and Rollings^7^, Lok *et al*.^8^, Makinde^9^, Maneschy *et al*.^10^, Qasim^11^ and Noor^12^. Also, of considerable interest for the present paper are the book by Bejan^13^, and the papers by Bejan^14^, and Khan and Gorla^15^. It should also be mentioned that the Bejan number was highlighted as a useful number for the topic of entropy generation analysis for non-Newtonian fluid (see Yazdi *et al*.^16^).

The ability of a third grade fluid model to describe the shear thinning and shear thickening properties for different kind of flows has inspired researchers to explore this model under many types of physical circumstances. Ellahi *et al*.^17^ obtained exact solutions for the generalized Couette flow problem in a third grade fluid. Abbasbandy and Hayat^18^ investigated the unsteady boundary layer flow of a special third grade fluid, and found that the boundary layer thickness increased with the third grade parameter and that suction reduced the boundary layer thickness. Kecebas and Yurusoy^19^ presented the unsteady two-dimensional boundary layer equations of a special third grade fluid and concluded that the non-Newtonian behaviour is directly proportional to the thickness of the boundary layer. Besides that, Ariel^20^ examined the steady flow of a third grade fluid in a porous flat channel and showed several sorts of solutions including an exact numerical solution, perturbation solution, an iterative solution and the approximate solutions. Later Hayat *et al*.^21^ reconsidered the problem of Ariel^20^ and found the three terms homotopy solution valid for all values of the third grade fluid.

Stagnation point exists on all solid figures moving in a fluid to freeze the fluid motion and this region confronts the peak of pressure, heat transfer and rates of mass deposition^22^. The pioneer work of Hiemenz^23^ in two-dimensional stagnation flow has attained much attention because of its utilization in engineering applications, especially in the polymer industry. The flow of an incompressible viscous fluid over a continuously stretching/shrinking surface is a dilemma faced in engineering processes with applications in industries, for instance wire drawing, plastic films drawing, glass-fibre production, crystal growth, and aerodynamic extrusion of plastic sheets. Sakiadis^24^ initiated the work on this topic throughout his investigation of the flow due to a continuously stretching surface from a slit into a stationary fluid with a constant speed. The flow was under Blasius type as the boundary layer thickness increased with the distance from the slit. Further, Crane^25^ extended the Sakiadis^24^ problem by considering the direct proportional relationship of the velocity to the distance from the slit and found an exact solution of the two-dimensional Navier-Stokes equations for a stretching sheet problem which is very useful to predict the system execution and hence contributed to the physical understandings of the relevant problems. On the other hand, Miklavcic and Wang^26^ investigated the properties of the flow due to a shrinking sheet with suction and concluded that sufficient suction on the surface are needed to sustain the flow over the shrinking sheet. Later, various literatures were presented regarding the stretching/shrinking surfaces under different states.

Mahapatra and Gupta^27^ obtained an exact similarity solution of the Navier-Stokes equations for the steady two-dimensional stagnation-point flow of an incompressible viscous fluid towards a stretching sheet and noticed that the thermal convection in the boundary layer occurred when the temperature of the stretching surface is constant. Wang^22^ studied the stagnation flow towards the shrinking sheet and found that the existence of a region filled with enduring opposite flow occured close to the surface where the transfers of heat, mass and momentum are blocked away from the sheet. Fang *et al*.^28^ presented an exact solution of the Navier-Stokes equation for the unsteady viscous flow over a continuously shrinking surface with mass suction problem. Futhermore, Hayat *et al*.^29^ investigated the mixed convection stagnation point flow and heat transfer over an unsteady stretching surface with the existence of a time-dependent free stream while Rohni *et al*.^30^ considered the unsteady flow over a continuosly shrinking surface with wall mass suction in nanofluid by using the Buongiorno’s model.

The present paper considers the similarity solutions of the unsteady stagnation-point flow and heat transfer of a special third grade fluid past a permeable stretching/shrinking sheet with the numerical solutions generated by a bvp4c function in Matlab software. The current results can be used to explain the characteristics and applications of non-Newtonian fluids in the field of tribology, automotive industry, etc. For instance, lubricating oils in the machineries are frequently tested for viscosity since it could affect the performance of oil and hence influence the lifespan of the equipment. As oils used for a long duration and still being utilized, factors including contamination particles and soot from incomplete combustion, can cause them to take on more non-Newtonian characteristics even at the lower shear rates. Thus, related parameters involved and appropriate situations such as stretching/shrinking surfaces, sufficient suction, etc. need to be applied and handled in order to control the non-Newtonian behaviour of the oil. To the best of our knowledge, this specific problem on special third grade fluid has not been considered before and therefore, the reported results are new and original.

## Problem Formulation

**Figure 1:**
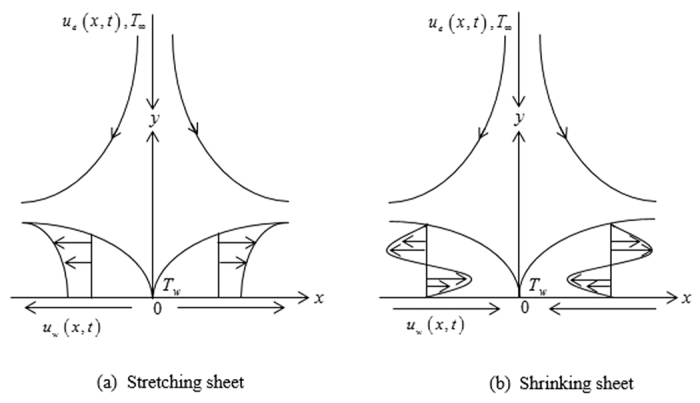
Physical model and coordinate system.

Consider the unsteady stagnation-point flow and heat transfer of a third grade fluid in the region *y* > 0 driven by an impulsively started stretching/shrinking surface, as shown in Fig. 1, where *x* and *y* are Cartesian coordinates measured along the surface and normal to it, respectively. It is assumed that at time *t* = 0 the surface starts to move with the velocity *U*_*w*_(*x*, *t*) = *λu*_*w*_(*x*, *t*) in an external free stream of velocity *u*_*e*_(*x*, *t*), where *λ* is a dimensionless constant with *λ* > 0 for a stretching surface and *λ* < 0 for a shrinking surface, respectively. It is also assumed that the mass flux velocity is *v*_*w*_(*t*) and the uniform temperature of the surface is *T*_*w*_, while that of the ambient fluid is *T*_∞_, where we consider that *T*_*w*_ > *T*_∞_ (heated surface). For third grade fluids, physical considerations were taken into account by Fosdick and Rajagopal^1^ in order to obtain the following form for the constitutive law


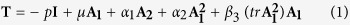


where **T** is the Cauchy stress tensor, *p* is the hydrostatic pressure, *μ* is the dynamic viscosity, **I** is the identity tensor and *α*_*i*_ (*i* = 1, 2) and *β*_*j*_ (*j* = 1, 2, 3) are material constants. Moreover, thermodynamics imposes the following constraints (see Fosdick and Rajagopal^1^):


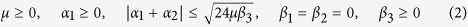


Under these assumptions, the basic equations of the problem under consideration can be written in Cartesian coordinates *x* and *y* as (see Abbasbandy and Hayat^18^)


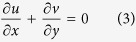



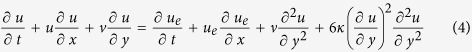



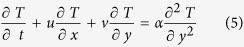


along with the boundary conditions


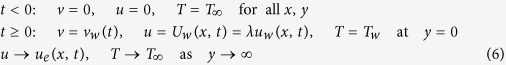


where *u* and *v* are the velocity components along the Cartesian coordinates *x* and *y*, *T* is the fluid temperature, *α* is the thermal diffusivity of the fluid, *ν* is the kinematic viscosity and *κ*(*x*, *t*) is the non-Newtonian parameter. It should be pointed out that for *κ*(*x*, *t*) = 0, equation (4) reduces to the case of viscous fluid. In order that equations (3) to (5), 4, 5 subject to the boundary conditions (6) admit similarity solutions, hence assume that


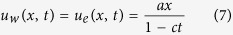


where *c* is a constant with dimension (*time*)^−1^ showing the unsteadiness of the physical problem. The effective stretching/shrinking rate 1/(1 − *ct*) increases or decreases with time since *c* > 0 or *c* < 0, respectively. Meanwhile, *κ* = *κ*_0_(1 − *ct*)^3^/*x*^2^ were assumed, where *κ*_0_ is a positive constant (see Mukhopadhyay and Andersson^31^).

The similarity solution of equations (3)–(5), 4, 5 of the following form will be explored


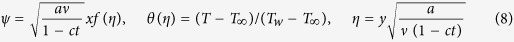


where *ψ* is the stream function defined in the usual form as *u* = ∂*ψ*/∂*y* and *v* = −∂*ψ*/∂*x*.

Thus,


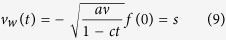


where *s* is the constant wall mass transfer parameter with *s* > 0 for suction and *s* < 0 for injection, respectively. Substituting (8) into equations (4) and (5), the following ordinary (similarity) equations will be obtained


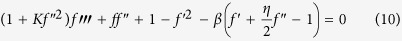



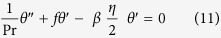


and the boundary conditions (6) become


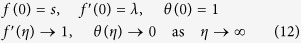


where primes denote differentiation with respect to *η*, Pr = *v*/*α* is the Prandtl number, *K* = 6*κ*_0_*a*^3^/*ν*^2^ is the constant dimensionless non-Newtonian parameter and *β* = *c*/*a* is the constant unsteadiness parameter with *β* > 0 for an accelerating flow and *β* < 0 for a decelerating flow, respectively. Here, only the case of *β* < 0 (see Fang *et al*.^28^) will be considered. It should be mentioned that for *K* = *β* = 0, equation (10) reduces to the classical stagnation-point flow problem first studied by Hermann^32^.

Quantities of interest in this problem are the skin friction coefficient *C*_*f*_ and the local Nusselt number *Nu*_*x*_, which are defined as


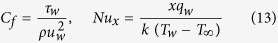


where *τ*_*w*_ is the skin friction along the surface of the stretching/shrinking sheet and *q*_*w*_ is the heat flux from the surface of the sheet, which are defined as


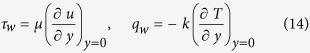


Substituting (8) into (14) and using (13), the following expression can be attained


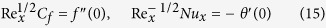


where 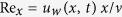 is the local Reynolds number.

## Stability Analysis

Merkin^33^ proved the existence of dual solutions and showed that stability analysis is the right approach, possibly holds for the steady-state problems, to recognize which of the solutions that is stable and hence physically applicable. Referring to Weidman *et al*.^34^, the presence of a dimensionless time variable, *τ* is necessary to determine which solution can be logically obtained in reality. Thus, variables (8) are modified by considering a dimensionless time variable, *τ* and the following new similarity variables are introduced:


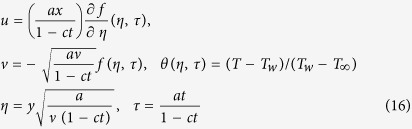


Substitute (16) into equations (4) and (5), the following equations can be obtained:


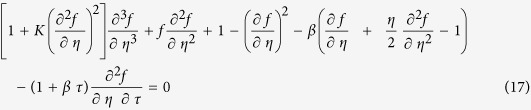



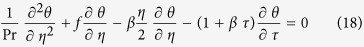


subject to the boundary conditions


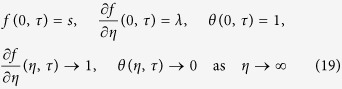


According to Weidman *et al*.^34^, take


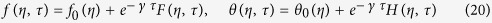


By substituting (20) into equations (17) and (18), the following equations are obtained:


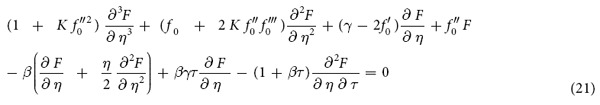



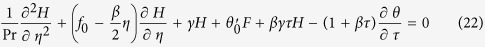


subject to boundary conditions


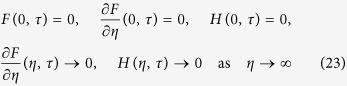


The stability of the steady stagnation-point flow and heat transfer equations (10) and (11) subject to the boundary conditions (12) has been studied by considering *τ* = 0 (see Weidman *et al*.^34^). Then, the following linear eigenvalue problem will be solved:


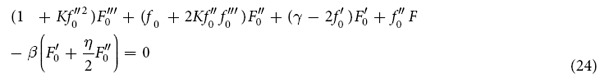



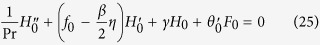


subject to boundary conditions


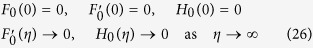


In order to determine the range of possible eigenvalues, one of the boundary conditions, namely 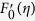 or *H*_0_(*η*) as *η* → ∞ should be relaxed (see Harris *et al*.^35^). The stability of the steady-state flow solution depends on the smallest eigenvalue, *γ*_1_. In this study, we relax the condition 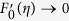 as *η* → ∞, and for a fixed value of the eigenvalue, *γ*, equations (24) and (25) will be solved with the introduction of new boundary condition that is 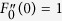.

## Results and Discussions

The ordinary differential equations (10)–(11), 11, subject to the boundary conditions (12), can be solved numerically using the bvp4c programme in Matlab software. In this study, the relative error tolerance was set to 10^−5^. The results from the numerical solution are reviewed in terms of the reduced skin-friction coefficient, *f* ″(0) and the reduced local Nusselt number, −*θ* ′(0) for different values of *s*, *K* and *λ*. This problem is considered under decelerating flow and Pr = 0.72 has been used where thermal diffusivity dominates the situation and controls the relative thickness of the momentum and thermal boundary layers. When Pr is small (Pr < 1), the heat diffuses immediately compared to the velocity (momentum), hence the thickness of the thermal boundary layer is much larger than the velocity of the boundary layer.

The numerical results for the reduced skin friction coefficient *f* ″(0) for the steady state flow obtained in this study via the bvp4c function in Matlab are compared with those of Wang^22^ and Bhattacharyya^36^ for validation purposes, as presented in Table 1. The comparisons are found to be in a very good agreement, and thus we are confident that the present numerical method is accurate. The accuracy of bvp4c function enables it to match with the numerical results that have been produced by using other methods such that Runge-Kutta method. From Tables 2, 3, 4, it can be seen that as the sheet is stretched, the values of *f* ″(0) drop and particularly when the sheet is stretched at *λ* = 1, *f* ″(0) becomes zero. This is because of the uniform movement of the fluid velocity with the surface of the boundary. Hence, there is no force that resists the motion of the fluid across the surface of the sheet (solid). Meanwhile, the value of −*θ* ′(0) increases when the sheet is stretched but decreases when the sheet is shrunk. As the sheet shrunk, there will be less space for the special third grade fluid to flow pass it and this reduces the convective heat transfer.

**Figure 2:**
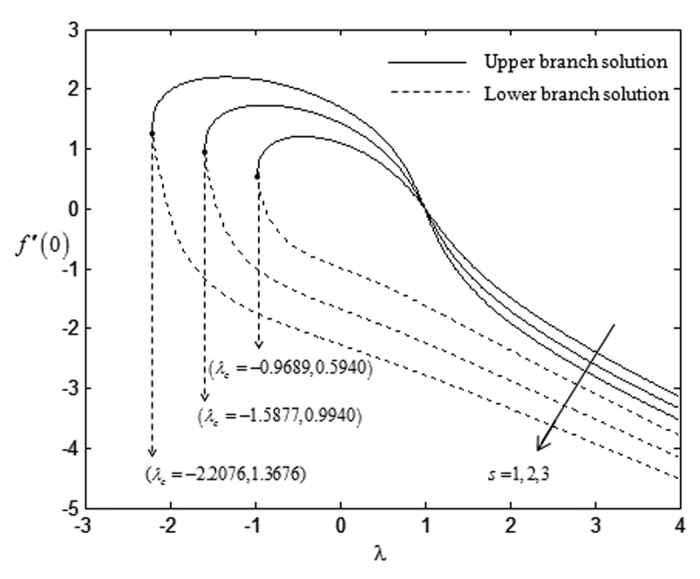
Variations of *f* ″(0) with *λ* for some values of *s* when *β* = −1 and *K* = 1.

**Figure 3:**
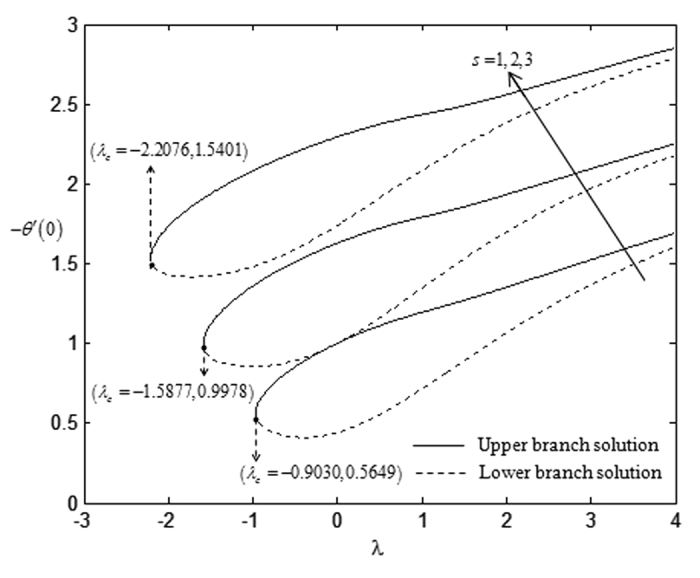
Variations of −*θ*′(0) with *λ* for some values of *s* when *β* = −1 and *K* = 1.

**Figure 4:**
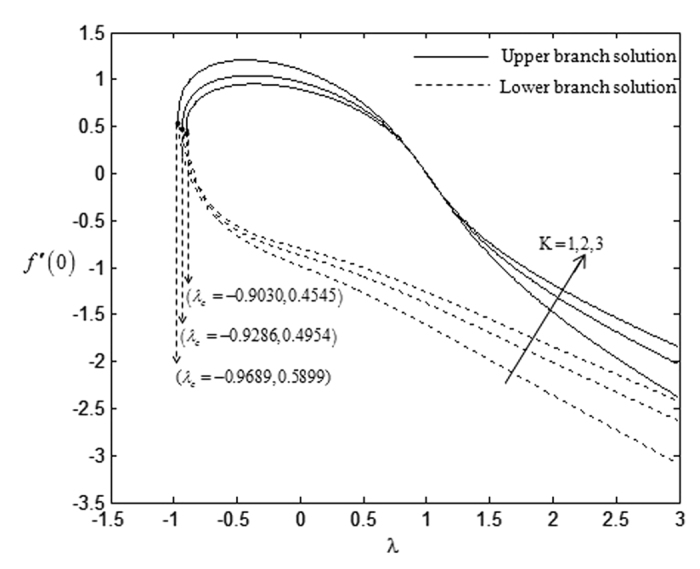
Variations of *f* ″(0) with *λ* for some values of *K* when *β* = −1 and *s* = 1.

**Figure 5:**
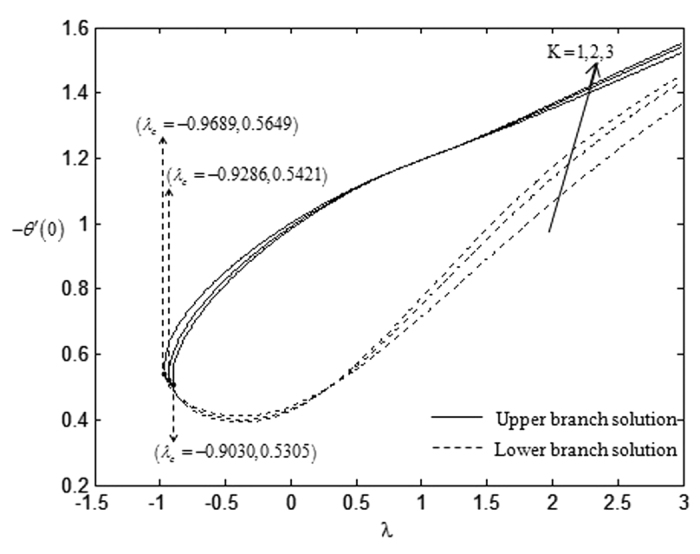
Variations of −*θ*′(0) with *λ* for some values of *K* when *β* = −1 and *s* = 1.

On the other hand, the effects of suction towards the critical values, *λ*_*c*_ which have been displayed in Table 5, express that the higher rate of suction *s* lowers the critical point values. However, this trend is in contrast with the impact of the non-Newtonian parameter *K* over the critical values. The higher non-Newtonian characteristics of the special third grade fluid seem to increase the critical point values and this is shown in Table 6. There is no solution when *λ* < *λ*_*c*_ and this statement is clearly illustrated in Figs 2, 3, 4, 5. The existence of dual solutions, namely first (upper branch) solution and second (lower branch) solution has been noticed from Figs 2, 3, 4, 5. It is seen that for *λ*_*c*_ < *λ* < 4 (see Figs 2 and 3), and for *λ*_*c*_ < *λ* < 3 (see Figs 4 and 5), the equations have two solutions, while for *λ* < *λ*_*c*_ < 4 (see Figs 2 and 3), and for *λ*_*c*_ < *λ* < 3 (see Figs 4 and 5), there is no solution, respectively. In this region, the full Navier-Stokes equations should be solved where *λ*_*c*_ is the critical value of *λ*. Moreover, Figs 2 and 3 indicate that the reduced skin friction coefficient, *f* ″(0) and the reduced local Nusselt number, −*θ* ′(0) will increase when the rate of suction increases. Figures 4 and 5 interpret that high non-Newtonian characteristics, (*K* = 3) on a special third grade fluid has small reduced skin friction coefficient and lower rate of heat transfer at the surface of the sheet compared to the case when *K* = 1 and *K* = 2.

**Figure 6:**
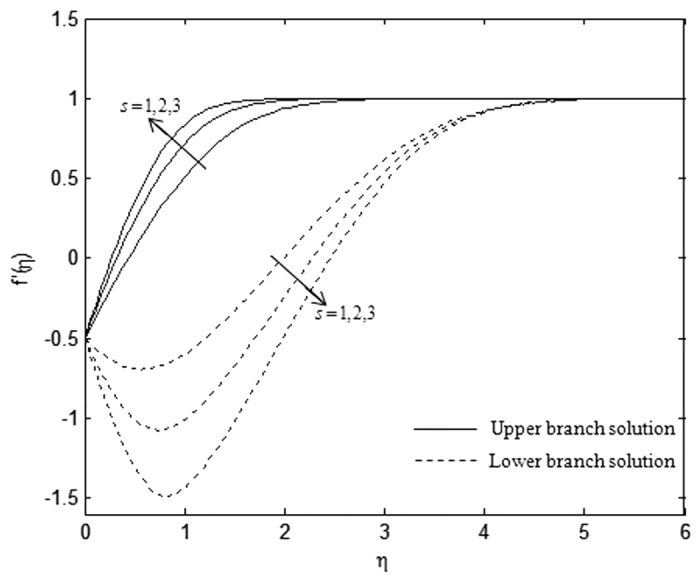
Velocity profiles, *f* ′(*η*) for different values of *s* when *λ* = −0.5, *β* = −1, and *K* = 1.

**Figure 7:**
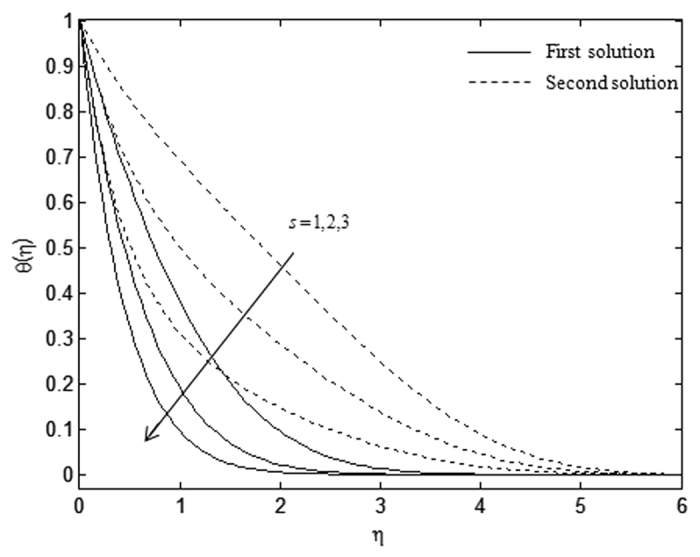
Temperature profiles, *θ*(*η*) for different values of s when *λ* = −0.5, *β* = −1, and *K* = 1.

**Figure 8:**
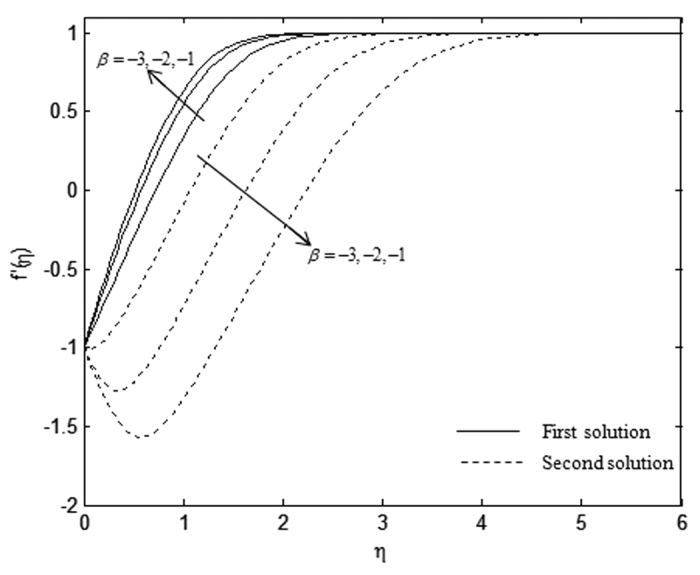
Velocity profiles, *f* ′(*η*) for different values of *β* when *λ* = −1, *s* = 3, and *K* = 1.

**Figure 9:**
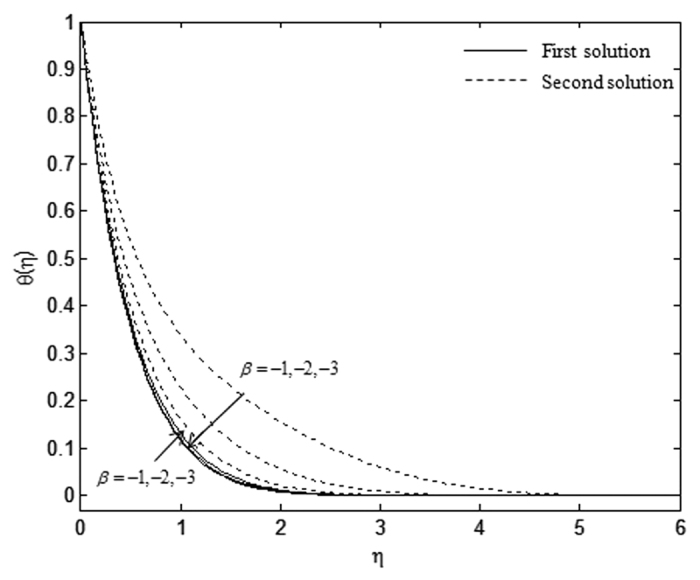
Temperature profiles, *θ*(*η*) for different values of *β* when *λ* = −1, *s* = 3, and *K* = 1.

**Figure 10:**
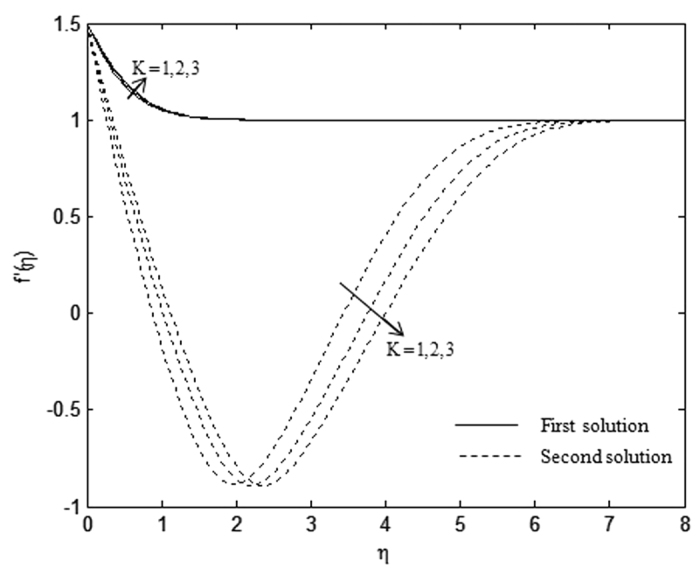
Velocity profiles, *f* ′(*η*) for different values of *K* when *λ* = 1.5, *s* = 1, and *β* = −1.

**Figure 11:**
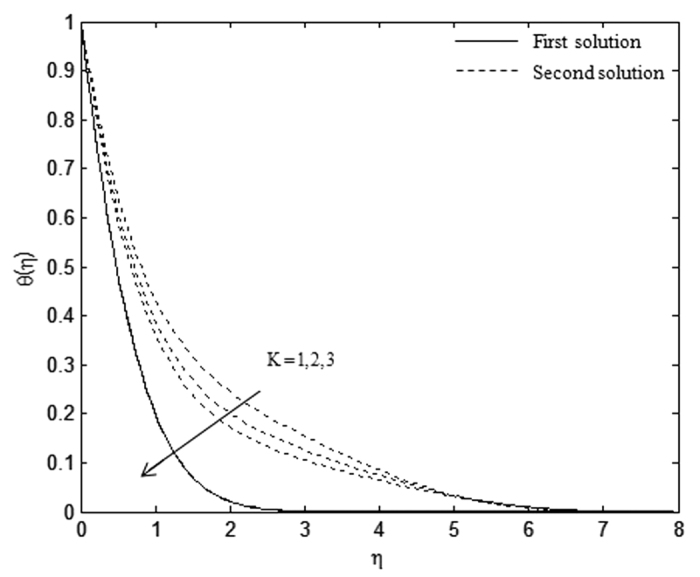
Temperature profiles, *θ*(*η*) for different values of *K* when *λ* = 0.5, *s* = 1, and *β* = −1.

**Figure 12:**
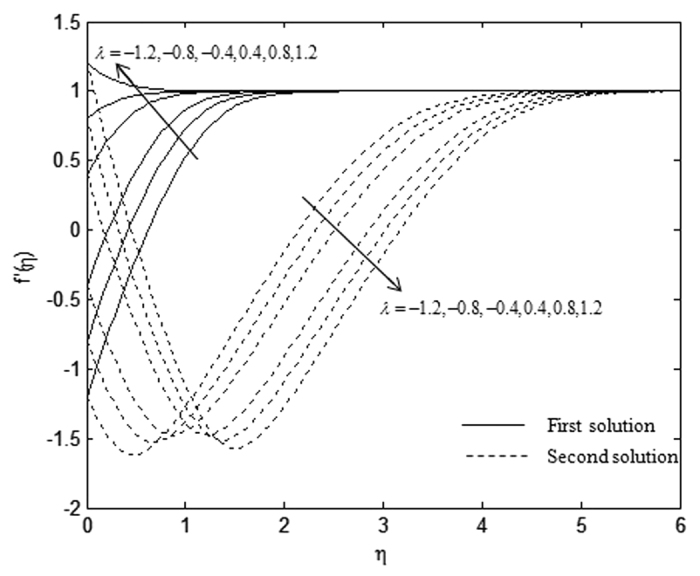
Velocity profiles, *f* ′(*η*) for different values of *λ* when *K* = 1, *s* = 3, and *β* = −1.

**Figure 13:**
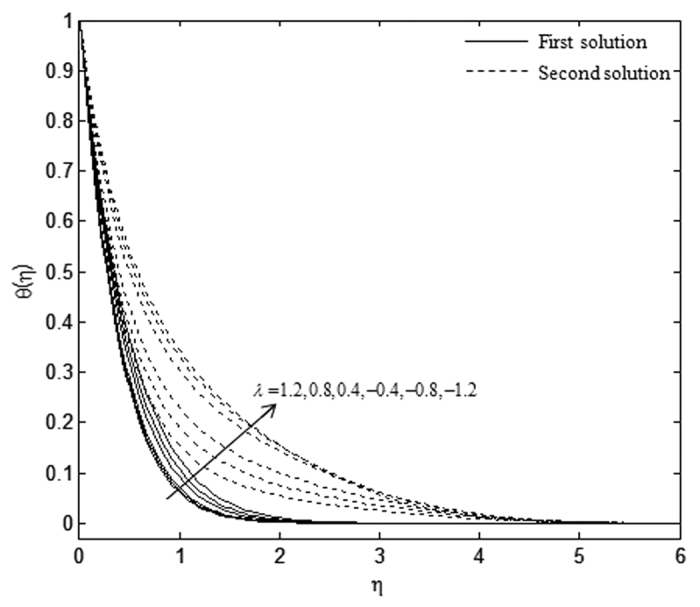
Temperature profiles, *θ(*η) for different values of *λ* when *K* = 1, *s* = 3, and *β* = −1.

The velocity and temperature profiles which have been shown in Figs 6, 7, 8, 9, 10, 11, 12, 13 satisfy the far field boundary conditions (10) asymptotically, which support the validity of the numerical results obtained and the existence of the dual solutions. For example, Figs 6, 8, 10 and 12 display the identified converged solutions when the plot of velocity profile *f* ′(*η*) approaches 1 as the boundary layer thickness value is less than or equals to 8 and they show the relationship *f*(0) = *s*. Figures 7, 9, 11 and 13 also able to reflect the boundary conditions *θ*(0) = 1 and *θ*(∞) → 0. In Figs 6 and 7, an increase in the rate of suction reduces the boundary layer thickness. Besides, the slower motion of the flow lowers the boundary layer thickness and this is shown in Figs 8 and 9. The high effects of non-Newtonian characteristic in the special third grade fluid will increase the boundary layer thickness and are well portrayed in Figs 10 and 11. Meanwhile, in Figs 12 and 13, the higher shrinking rate increases the boundary layer thickness, but the second solution opposes the trend where the higher shrinking rate decreases the boundary layer thickness compared to the stretching case. As mentioned earlier in this paper, the existence of dual solutions when the sheet is stretched and shrunk as the value of *λ* lies in between *λ*_*c*_ < *λ* < 4 have been noticed. Therefore, there is a necessity to conduct the stability analysis and we found that the first solution (upper branch) is stable and physically applicable while the second solution (lower branch) is unstable. The stable solution is identified based on the positive smallest eigenvalue whereas the unstable solution is recognized based on the negative smallest eigenvalue. Table 7 illustrates the smallest eigenvalue, *γ*_1_ for some values of *λ* when *s* = 3, *K* = 3, and *β* = −1.

## Conclusions

This paper considered numerical solutions and stability analysis of the unsteady stagnation-point flow and heat transfer of a special third grade fluid past a permeable stretching/shrinking sheet. From this study, the reduced skin friction coefficient and the reduced local Nusselt number at the sheet increased as the rate of suction increased. Higher non-Newtonian characteristics of a special third grade fluid has smaller reduced skin friction coefficient and lower rate of heat transfer at the surface of the sheet compared to lower non-Newtonian characteristics of a special third grade fluid. Dual solutions can be obtained when the sheet is stretched and shrunk when *λ*_*c*_ < *λ* ≤ 4. Therefore, stability analysis has been done to show that the first solution (upper branch) is stable, whereas the second solution (lower branch) is unstable.

## Additional Information

**How to cite this article**: Naganthran, K. *et al*. Unsteady stagnation-point flow and heat transfer of a special third grade fluid past a permeable stretching/shrinking sheet. *Sci. Rep*. **6**, 24632; doi: 10.1038/srep24632 (2016).

## Figures and Tables

**Table 1:** Comparison of numerical results for the values of *f* ″(0) for the steady-state flow when the surface is shrunk and when *K* = 0, *β* = 0, *s* = 0 and Pr = 0.72.

*λ*	Present results		Bhattacharyya^32^		Wang^22^	
	First solution	Second solution	First solution	Second solution	First solution	Second solution
−0.25	1.4022407	–	1.4022405	–	1.40224	–
−0.50	1.4956697	–	1.4956697	–	1.49567	–
−0.75	1.4892982	–	1.4892981	–	1.48930	–
−1.00	1.3288168	0	1.3288169	0	1.32882	0
−1.15	1.0822311	0.1167020	1.0822316	0.1167023	1.08223	0.116702
−1.20	0.9324733	0.2336496	0.9324728	0.2336491	–	–
−1.2465	0.5842759	0.5542976	0.5842915	0.5542856	0.55430	–
−1.24657	0.5745397	0.5640169	0.5745268	0.5639987	–	–

**Table 2:** Dual solutions of *f* ″(0) and −*θ*′(0) for the shrinking surface (*λ* < 0) and stretching surface (*λ* > 0) when *s* = 3, *K* = 1 and *β* = −1.

*λ*	*f* ″(0)		−θ′(0)	
	First solution	Second solution	First solution	Second solution
−1.5	2.193377	−1.28285	1.938451	1.423448
−1.0	2.166659	−1.72211	2.081987	1.479233
−0.5	2.004485	−2.01423	2.197901	1.586895
0	1.704967	−2.26468	2.294649	1.736056
0.5	1.185006	−2.51231	2.374083	1.907967
1.0	0	−2.77119	2.433741	2.081403
1.5	−1.2484	−3.04334	2.490572	2.241382

**Table 3:** Dual solutions of *f* ″ (0) and −*θ*′(0) for the shrinking surface (*λ* < 0) and stretching surface (*λ* > 0) when *s* = 3, *K* = 3 and *β* = −1.

*λ*	*f* ″(0)		−θ′(0)	
	First solution	Second solution	First solution	Second solution
−1.5	1.496961	−0.776316	1.854743	1.465027
−1.0	1.555887	−1.190537	2.032096	1.515417
−0.5	1.476845	−1.413027	2.166841	1.641006
0	1.288081	−1.592146	2.277386	1.821447
0.5	0.941126	−1.771238	2.368117	2.020089
1.0	0	−1.964797	2.433741	2.202279
1.5	−0.991262	−2.173651	2.496140	2.354546

**Table 4:** Dual solutions of *f* ″(0) and −*θ*′(0) for shrinking surface (*λ* < 0) and stretching surface (*λ* > 0) when *s* = 3, *K* = 1 and *β* = −3.

λ	*f* ″(0)		−θ′(0)	
	First solution	Second solution	First solution	Second solution
−1.5	1.630754	−0.151577	1.892278	1.737387
−1.0	1.897845	−1.431371	2.068307	1.744285
−0.5	1.835419	−1.898569	2.193046	1.806175
0	1.594179	2.221268	2.293849	1.904763
0.5	1.117490	−2.498897	2.375587	2.026513
1.0	0	−2.765888	2.437028	2.157663
1.5	−1.191628	−3.036448	2.495190	2.287105

**Table 5:** Critical values of *λ* for various values of *s* when *β* = −1 and *K* = 1.

*s*	*λ*_*c*_
1	−0.9689
2	−1.5877
3	−2.2076

**Table 6:** Critical values of *λ* for various values of *K* when *β* = −1 and *s* = 1.

K	λ_c_
1	−0.9689
2	−0.9286
3	−0.9030

**Table 7:** Smallest eigenvalue, *γ*_1_ for some values of *λ* when *s* = 3, *K* = 3, and *β* = −1.

*λ*	First solution (Upper branch), *γ*_1_	Second solution (Lower branch), *γ*_1_
−1.5	0.0968	−0.0237
−1.0	0.3137	−0.1873
−0.5	0.4553	−0.2917
0	0.5000	−0.3203
0.5	0.5770	−0.4029
1.0	0.5900	−0.5230
1.5	0.6449	−0.6055
